# Supplementary material for: Gamete dimorphism of the isogamous green alga (Chlamydomonas reinhardtii), is regulated by the mating type-determining gene, MID
Source: Commun Biol. 2022 Dec 6;5:1333. doi: 10.1038/s42003-022-04275-y (PMC9726906; doi:10.1038/s42003-022-04275-y)
Supplement: Supplementary file 2 — Supplementary Information [file 42003_2022_4275_MOESM2_ESM.pdf]

Gamete Dimorphism of the Isogamous Green Alga (*Chlamydomonas reinhardtii*), is Regulated by the Mating Type-Determining Gene, *MID*

Ryoya Innami[1], Shinichi Miyamura[1], Masako Okoshi[1], Tamotsu Nagumo[2], Kensuke Ichihara[3], Tomokazu Yamazaki[4], Shigeyuki Kawano[4]

[1]Faculty of Life and Environmental Sciences, University of Tsukuba, Tsukuba, Ibaraki, 305 8572 Japan, [2]Echigo Natural History Laboratory, Ojiya, Niigata 947-0041, Japan, [3]Field Science Center for Northern Biosphere, Hokkaido University, Funami-cho, Muroran 051-0013, Japan, [4]Graduate School of Frontier Sciences, The University of Tokyo, Wakashiba, Kashiwa, Chiba, 277-0871 Japan

Corresponding author: Shinichi Miyamura
Email: miyamura.shinichi.fw@u.tsukuba.ac.jp

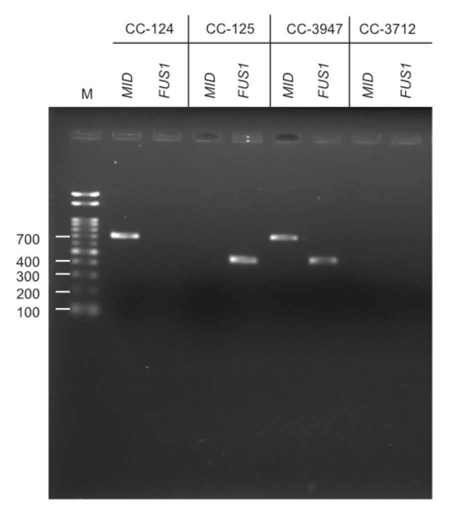

Supplementary Figure 1. PCR analysis of the mating type genotype of wild-type and sex-reversed strains

Presence or absence of mating type-specific genes (*MID* and *FUS1*) was analysed in the wild-type mt^{-} (CC-124), mt^{+} (CC-125), and sex-reversed strains (CC-3947 and CC-3712). Figure shows an uncropped gel image. *M*: marker (bp) (Gene Ladder 100, Nippon Gene Co., Ltd.).

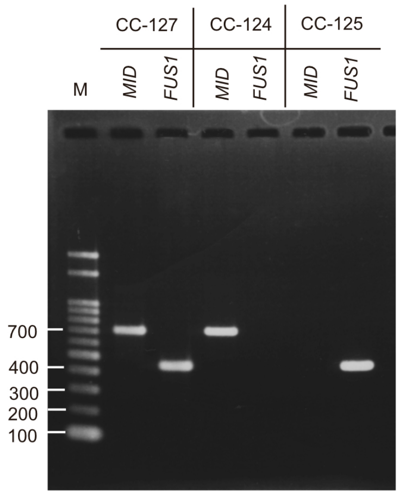

Supplementary Figure 2. PCR analysis of the mating type genotype of diploid strains

Presence or absence of mating type-specific genes (*MID* and *FUS1*) was analysed in diploid strains (CC-127), wild-type mt^{-} (CC-124), and mt^{+} (CC-125). Figure shows an uncropped gel image. *M*: marker (bp) (Gene Ladder 100, Nippon Gene Co., Ltd.).

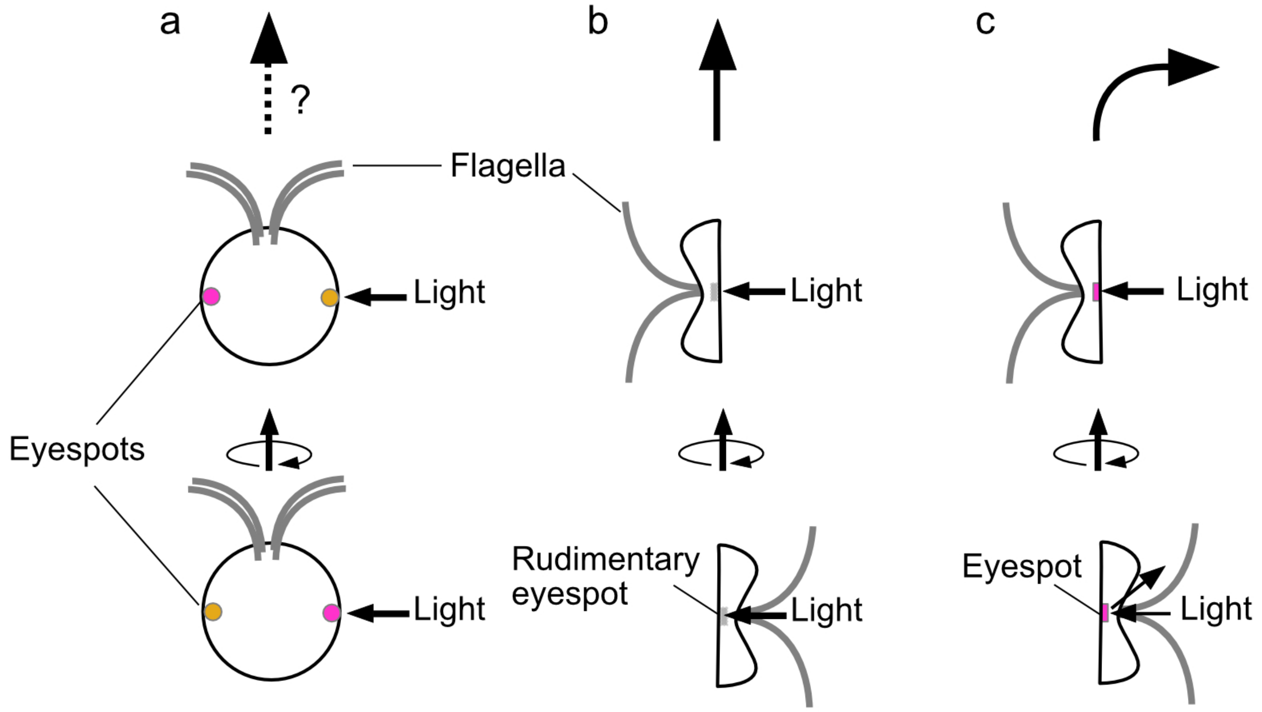

Supplementary Figure 3. Schematic drawing of the eyespot arrangement and phototactic movement of the abnormal planozygote of *C. reinhardtii*, eyeless and wild-type strain of *Mesostigma viride*

a Abnormal planozygote of *C. reinhardtii*. **b** Eyeless strain of *Mesostigma viride*. **c** Wild-type strain of *M. viride*. In (**a**) and (**b**), the cell reacts to light stimuli twice per rotation, while in (**c**) the cell reacts once per rotation. The large dotted line and solid arrows indicate the predicted and actual swimming direction after light stimulation, respectively. Eyespot arrangement and phototaxis of *C. reinhardtii* and *M. viride* are drawn based on Fig. 6b and c in this study and Fig. 9 of Matsunaga et al. [1], respectively.

Supplementary Table 1. Mating type/sex, MSP and phototaxis of chlorophyte gametes and planozygotes.

Class / Order / Species	Sexual reproduction type	Cell type	Mating type / sex*	MSP (α/β)	Number of flagella /cell	Number of eyespot(s) /cell	Side-by-side alignment of two eyespots	Coordinated alignment of flagella and eyespot(s)	Phototaxis	Reference
Chlorophyceae										
Chlamydomonadales										
Chlamydomonas reinhardtii	Isogamy	Gamete	Minus	β	2	1		Yes**	Positive	
		Gamete	Plus	α	2	1		Yes	Positive	This study, 2
		Planozygote			4	2	Yes	Yes	Negative	
Ulvophyceae										
Dasycladales										
Acetabularia caliculus	Isogamy	Gamete	Minus	α	2	1		Yes	Positive (weak)	
		Gamete	Plus	β	2	1		Yes	positive (weak)	3, 4, 5
		Planozygote			4	2	Yes	Yes	ND (inactive)	
Bryopsidales										
Bryopsis maxima	Marked anisogamy	Gamete	Male	ND	2	0		ND	ND	
		Gamete	Female	β	2	1		Yes	Positive	6, 7
		Planozygote			4	1	No	Yes	Negative	
Ulvales										
Ulva prolifera	Slight anisogamy	Gamete	Minus	α	2	1		Yes	Positive	
		Gamete	Plus	β	2	1		Yes	Positive	8, 9
		Planozygote			4	2	Yes	Yes	Negative	
U. partita	Slight anisogamy	Gamete	Minus	α	2	1		Yes	Positive	
		Gamete	Plus	β	2	1		Yes	Positive	10, 11
		Planozygote			4	2	Yes	Yes	Negative	

U. arasakii	Anisogamy	Gamete	Male	α	2	1		Yes	Positive	
		Gamete	Female	β	2	1		Yes	Positive	12, 13
		Planozygote			4	2	Yes	Yes	Negative	
Ulotrichales										
Monostroma nitidum	Isogamy	Gamete	Minus	β	2	1		Yes	Positive	
		Gamete	Plus	α	2	1		Yes	Positive	14, 15
		Planozygote			4	2	Yes	Yes	Negative	
M. angicava	Anisogamy	Gamete	Male	α	2	1		Yes	Positive	
		Gamete	Female	β	2	1		Yes	Positive	16, 17
		Planozygote			4	2	Yes	Yes	Negative	
Collinsiella cava	Slight anisogamy	Gamete	Male	α	2	1		Yes	Positive	
		Gamete	Female	β	2	1		Yes	Positive	18, 19
		Planozygote			4	2	Yes	Yes	Negative	
Nephroselmidophyceae										
Nephroselmidales										
Nephroselmis olivacea	Isogamy	Gamete	Minus	α	2	1		Yes	ND	
		Gamete	Plus	β	2	1		Yes	ND	20
		Planozygote (swimming pair)			4	> 1	ND	ND	ND	

ND, not determined. *Mating types and sexes were designated according to the original description. **Eyespot(s) occupy a specific position in the cell relative to the flagellar beat plane.

Supplementary Table 2. Strains used in this study.

Strain	Name	Genotype	Source	Reference
CC-124	wild-type 137c mt^{-}	*nit1 nit2 agg1* mt^{-}	Chlamydomonas Resource Center	
CC-125	wild-type 137c mt^{+}	*nit1 nit2* mt^{+}	Chlamydomonas Resource Center	
CC-127	wild-type diploid	*arg2/arg7* 2N mt^{-}	Chlamydomonas Resource Center	21
CC-3712	mid deletion (Beck CF181)	*mid* mt^{-}	Chlamydomonas Resource Center	22
CC-3947	K33	*nic7 thi10* with a *MID* transgene mt^{+} *T-MID*	Chlamydomonas Resource Center	23

References

1. Matsunaga, S., Watanabe, S., Sakaushi, S., Miyamura, S. & Hori, T. Screening effect diverts the swimming directions from diaphototactic to positive phototactic in a disk-shaped green flagellate *Mesostigma viride*. *Photochem. Photobiol* **77**, 324-332 (2003).
2. Adams, G. M. W. Effect of sunlight on inheritance of chloroplast genes in *Chlamydomonas reinhardtii*. *Genetics* **80**, s8-s9 (1975).
3. Miyamura, S. & Nagumo, T. Sex-specific cell fusion pattern of isogametes in marine green alga, *Acetabularia caliculus* (Ulvophyceae, Chlorophyta). *Cytologia* **81**, 1-5 (2016).
4. Arasaki, S. On the life history of *Acetabularia calyculus* Quoy et Gaimard. *Bot Mag Tokyo* **56**, 383-391 (1942).
5. Sano, O., Ikemori, M. & Arasaki, S. Distribution and ecology of *Acetabularia calyculus* along the coast of Noto Peninsula. *Jpn. J. Phycol.* **29**, 31-38 (1981).
6. Miyamura, S., Sakaushi, S., Hori, T., Mitsuhashi, F. & Nagumo, T. Sex-specific cell surface structure of anisogametes: Morphological changes during fertilization of *Bryopsis maxima* (Ulvophyceae, Chlorophyta) revealed by ultra-high-resolution field emission SEM. *J. Phycol.* **41**, 114–125 (2005).
7. Tatewaki, M. Culture of *Bryopsis* and isolation and culture of protoplasts. In *Methods in Phycological Studies* (eds. Nishizawa, K. & Chihara, M.) 133-142 (Kyoritsu Shuppan Co. Ltd., 1979).
8. Ichihara, K., Yamazaki, T., Miyamura, S., Hiraoka, M. & Kawano, S. Asexual thalli originated from sporophytic thalli via apomeiosis in the green seaweed *Ulva*. *Sci. Rep.* **9**, 913523 (2019).
9. Hiraoka, M., Dan, A., Shimada, S., Hagihira, M., Migita, M. & Ohno, M. Different life histories of *Enteromorpha prolifera* (Ulvales, Chlorophyta) from four rivers on Shikoku island, Japan. *Phycologia* **42**, 275-284 (2003).
10. Mogi, Y., Kagami, Y., Kuwano, K., Miyamura, S., Nagumo, T. & Kawano, S. Asymmetry of eyespot and mating structure positions in *Ulva compressa* (Ulvales, Chlorophyta) revealed by a new field emission scanning electron microscopy method. *J. Phycol.* **44**, 1290-1299 (2008).
11. Ichihara, K., Suzuki, R., Yamazaki, T., Ota, S., Mogi, Y., Kagami, Y., Kuwano, K. & Kawano, S. *Ulva partita* sp. nov., a novel *Enteromorpha*-like *Ulva* species from Japanese coastal areas. *Cytologia* **80**, 261-270 (2015).
12. Miyamura, S., Hori, T. & Nagumo, T. Eyespot behavior during the fertilization of gametes in *Ulva arasakii* Chihara (Ulvophyceae, Chlorophyta). *Phycol. Res.* **51**, 143-146 (2003).
13. Chihara, M. *Ulva arasakii*, A new species of green algae: its life history and taxonomy. *Bull. Nat. Sci. Mus. Tokyo* **12**, 849-862 (1969).
14. Miyamura, S., Nagumo, T., Maegawa, M. & Hori, T. Rearrangement of the flagellar apparatuses and eyespots of isogametes during the fertilization of the marine green alga, *Monostroma nitidum* (Ulvophyceae, Chlorophyta). *Phycol. Res*. **63**, 284-299 (2015).

15. Kida, W. Studies on the morphology and ecology of *Monostroma* in Ise Bay and vicinity, Japan. *J. Fac. Pref. Univ. Mie* **7**, 81-164 (1967).

16. Miyamura, S., Nakayama, T., Mitsuhashi, F., Nagumo, T, Sato, T., Motomura, T. & Hori, T. Sex-specific positioning of the mating structure in scale bearing gametes of *Monostroma angicava* and *Collinsiella cava* (Ulvophyceae, Chlorophyta): A possible widespread difference between male and female gametes. *J. Phycol.* **57**, 510-527 (2021).

17. Togashi, T., Motomura, T. Ichimura, T. & Cox P A. Gametic behavior in a marine green alga, *Monostroma angicava*: an effect of phototaxis on mating efficiency. *Sex. Plant. Reprod.* **12**, 158-163 (1999).

18. Nakayama, T. & Inouye, I. Ultrastructure of the biflagellate gametes of *Collinsiella cava* (Ulvophyceae, Chlorophyta). *Phycol. Res*. **48**, 63–73 (2000).

19. Chihara, M. *Collinsiella* in Japan, with special reference to the life-history. *Sci. Rep. TKD. Sect. B* **9**, 181-198 (1960).

20. Suda, S., Watanabe, M. M. & Inouye, I. Electron microscopy of sexual reproduction in *Nephroselmis olivacea* (Prasinophyceae, Chlorophyta). *Phycol. Res*. **52**, 273-283 (2004).

21. Ebersold, W. T. *Chlamydomonas reinhardti*: Heterozygous diploid strains. *Science* **157**, 447-449 (1967).

22. Ferris, P. J., Armbrust, E. V. & Goodenough, U. W. Genetic structure of the mating-type locus of *Chlamydomonas reinhardtii*. *Genetics* **160**, 181-200 (2002).

23. De Hoff, P. L., Ferris, P., Olson, B. J. S. C., Miyagi, A., Geng, S. & Umen, J. G. Species and population level molecular profiling reveals cryptic recombination and emergent asymmetry in the dimorphic mating type locus of *C. reinhardtii*. *PLoS Genet.* **9**, e1003724 (2013).
